# Anti-Colorectal Cancer Effects of *Inonotus hispidus* (Bull.: Fr.) P. Karst. Spore Powder through Regulation of Gut Microbiota-Mediated JAK/STAT Signaling

**DOI:** 10.3390/nu14163299

**Published:** 2022-08-12

**Authors:** Hongxin Yang, Siyu Li, Yidi Qu, Lanzhou Li, Yu Li, Di Wang

**Affiliations:** 1 School of Life Sciences, Jilin University, Changchun 130012, China; 2 Engineering Research Center of Chinese Ministry of Education for Edible and Medicinal Fungi, School of Plant Protection, Jilin Agricultural University, Changchun 130118, China

**Keywords:** *Inonotus hispidus*, CRC, gut microbiota, serum metabolomics, JAK/STAT

## Abstract

*Inonotus hispidus* (Bull.: Fr.) P. Karst. spore powder (IHS) contains polyphenols and triterpenoids with pharmacological effects. Here, we analyzed its composition, and we investigated the effects of IHS on colorectal cancer (CRC) in B6/JGpt-*Apc^em1Cin(min)^*/Gpt (*Apc^Min/+^*) mice and its potential mechanisms by analyzing gut microbiota and serum metabolomics. The enzyme-linked immunosorbent assays and Western blotting were used to confirm the changes in the cytokine and protein levels associated with IHS administration. The IHS affected the abundance of gut microbiota and the level of *L*-arginine (*L*-Arg). Furthermore, the IHS influenced T cells in *Apc^Min/+^* mice by increasing the interleukin (IL)-2 and decreasing the IL-5, -6, and -10 levels, thus suppressing tumor development. Overall, IHS showed anti-CRC properties in *Apc^Min/+^* mice by affecting the gut microbiota and serum metabolites, which in turn affected the Janus kinase (JAK)/signal transducer and activator of transcription (STAT) signaling, and regulated the abundance of CD8^+^ T cells. These results provide experimental support for the potential future treatment of CRC with IHS.

## 1. Introduction

As the second leading cause of cancer deaths, 1.9 million new cases of colorectal cancer (CRC) (including anal cancer) and 935,000 CRC-related deaths occurred in 2020, worldwide [1]. The patients with CRC may be susceptible to coronavirus disease (COVID-19) [2]. Genetics, unhealthy living and eating habits, such as a high-fat and low-fiber diet, are the main predisposing factors for CRC [3,4]. Fiber can be fermented by the gut microbiota to produce short-chain fatty acids, which positively affect the immune system and lower the risk of CRC [4]. The changes in the composition of the gut microbiota may be important etiological factors in CRC development and progression [5]. In cases with imbalanced gut microbiota, the increased secretion of bacterial toxins and carcinogenic secondary metabolites impairs the gut barrier-functions, causing immune dysregulation, which can lead to CRC [6]. The T lymphocyte-mediated immune functions play important roles in CRC [7]. In particular, CD8^+^ T cell infiltration is independently favorable for CRC prognosis [8]. The CRC patients with high levels of CD8^+^ T cells at the center of the tumor or at the invasive margins typically show a longer survival time [9].

*Inonotus hispidus* (Bull.: Fr.) P. Karst. is a medicinal fungus of the family Hymenochaetaceae and is the source of the traditional Chinese herb “Sanghuang” [10]. *I. hispidus* was listed in the ancient Chinese book *The Herbal Classic of Shen Nong* as a treatment for gynecological diseases. In modern research, *I. hispidus* has been artificially cultivated by Chinese scientists [10] and was shown to contain physiologically active triterpenes and polyphenols [11]. *I. hispidus* substrates and their extracts have immunomodulatory [12], anti-tumor [13], antibacterial [14], and antioxidant [15] properties. In H22 tumor-bearing mice, *I. hispidus* solid fermentation powder can inhibit tumor development by regulating immune functions [13]. In humans, *I. hispidus* extract enhances T cell activation while apoptosis occurs [12]. However, the current studies have focused on *I. hispidus* substrates and their extracts, whereas *Inontus hispidus* (Bull.: Fr.) P. Karst. spore powder (IHS) has received less attention, thus its composition and effects with respect to CRC are unclear.

The B6/JGpt-*Apc^em1Cin(min)^*/Gpt (*Apc^Min/+^*) mice were used as the CRC models, due to an adenomatous polyposis coli (*Apc*) mutation. The *Apc* gene is a colorectal cancer oncogene, and in over 70% of the CRC cases it shows mutations which lead to the constitutive activation of the Wnt/β-catenin pathway in the intestinal epithelial cells [16,17]. The signal transducer and activator of transcription 3 (STAT3) promotes the initial stages of CRC formation in *Apc^Min/+^* mice [18]. The activated Janus kinase (JAK)/STAT pathway observed in most solid tumors can affect CRC development by modulating the cell growth, survival, invasion, and migration [19], and some of its antagonists impede the progression of malignancies [20]. The interleukins (IL)-2 and -6 are the essential mediators of T cell differentiation and function during immune responses related to the activation of the JAK/STAT pathway [21,22]. In our previous study, calf thymus polypeptides effectively increased the IL-2 and CD8^+^ T cell levels to inhibit CRC growth in *Apc^Min/+^* mice [23]. Similarly, the knockout of the monocyte chemo-attractant protein 1 in *Apc^Min/+^* mice increased the abundance of CD8^+^ T cells and decreased the IL-6 expression, thus alleviating the development of CRC [24].

In this study, the suppressive effects of IHS on CRC development in *Apc^Min/+^* mice were confirmed. According to the gut microbiota and serum metabolomics, the IHS treatment restored the imbalance in the gut microbiota and the levels of serum metabolites, which then regulated the abundance of CD8^+^ T cells related to JAK/STAT signaling.

## 2. Materials and Methods

### 2.1. IHS Component Analysis

The IHS was collected in Linqing Sanghuang Town (Linqing, China) and was identified by Prof. Y. Li from Jilin Agriculture University.

#### 2.1.1. Nutrients

The Kjeldahl method was used to measure the total nitrogen content, and a conversion factor of 6.25 was used to calculate the total protein content [25]. The phenol sulfate method was used to determine the total sugar content. Fat was extracted from the dried IHS samples using petroleum ether and a Soxhlet apparatus, and the fat content was assessed, using the residual method [25]. The gravimetric method was used to assess the total dietary fiber content [25], and the total polyphenols [25], alkaloids [26], flavonoids [26], sterols [27], triterpenoids [28], and saponins [29] were assessed using UV spectrophotometry.

#### 2.1.2. Minerals and Heavy Metals

The IHS (2 g) was placed in a polytetrafluoroethylene digestion tank, and 5 mL nitric acid was added. After digestion, the samples were washed using ultrapure water and were diluted to the mark. The concentrations of calcium (Ca), sodium (Na), potassium (K), Arsenic (As), lead (Pb), mercury (Hg), cadmium (Cd), copper (Cu), chromium (Cr), iron (Fe), zinc (Zn), selenium (Se), and manganese (Mn) were analyzed using an inductively coupled plasma mass spectrometry device (iCAPQ; Thermo Fisher Scientific, Waltham, MA, USA) [30].

### 2.2. Animal Experimental

All of the procedures were approved by the Institutional Animal Ethics Committee of Jilin University (SY202103008), and all of the experimental procedures involving animals were performed in strict accordance with the institutional guidelines. Eight-weeks-old *Apc^Min/+^* mice purchased from GemPharmatech Co., Ltd., Jiangsu, China, (SCXK [SU] 2018-0008) were housed in a controlled environment at 23 ± 1 °C and 50–60% humidity under a 12/12 h light/dark cycle. A rodent diet with 60 kcal% fat (#D12492; Research Diet, New Brunswick, NJ, USA) and water were provided ad libitum.

**Figure 1:**
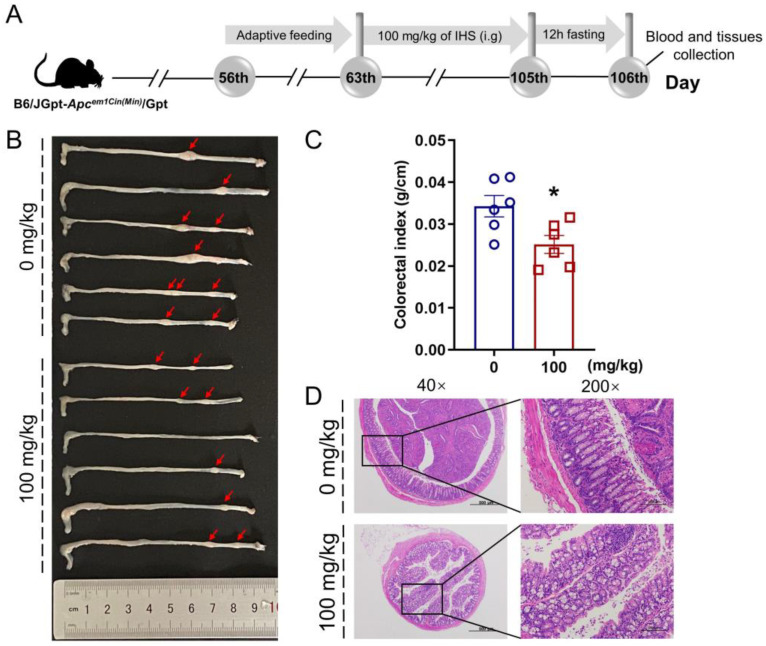
IHS inhibits the growth of CRC in *Apc^Min/+^* mice. (**A**) Animal experiments and agent treatment process; (**B**) Representative photographs of the colorectum of vehicle- and IHS-treated *Apc^Min/+^* mice (*n =* 6, each); (**C**) IHS reduced colorectal indexes (*n =* 6). Data were analyzed using a one-way ANOVA and are shown as means ± standard error of the man (SEM); * *p* < 0.05 vs. vehicle-treated *Apc^Min/+^* mice; (**D**) Pathological examination of colorectal tissue through hematoxylin and eosin staining (40×, 500 μm; 200×, 100 μm).

After one week of adaptive feeding, twelve *Apc^Min/+^* mice were randomly assigned to two groups (*n =* 6, each), and 100 mg/kg IHS or vehicle only was orally administered for six weeks. The standard procedures of intragastric gavage were on individuals of both groups to account for the effects of gavage stress. The body weights were monitored weekly. After the last administration, *Apc^Min/+^* mice were fasted for 12 h, and blood samples were collected from the tail vein. The mice were killed through intraperitoneal injection with 150 mg/kg sodium pentobarbital, after which the feces were collected from the cecum in a sterile environment. The organs including the colorectum, heart, liver, spleen, lungs, and kidneys were collected for organ index calculations and biochemical and pathological assessment (Figure 1A).

### 2.3. Flow Cytometry

The blood samples were stained at 4 °C for 30 min in the dark, using APC anti-mouse CD45 (#103112), FITC anti-mouse CD3 epsilon (CD3e; #100306), PE anti-mouse CD4 (#100408), PE anti-mouse CD8a (#100708), and PE anti-mouse CD19 (#115508) antibodies. The FITC Armenian Hamster IgG isotype (#400905), PE Rat IgG2b (#400607), PE Rat IgG2a (#400507), and APC Rat IgG2b (#400611) were used as the isotype controls. The non-nucleated cells were removed, using red blood cell lysis buffer (Gibco BRL, San Francisco, CA, USA) according to the manufacturer’s instructions. After centrifugation, the collected cells were washed using PBS and were analyzed through flow cytometry (CytoFLEX, Beckman Coulter, Brea, CA, USA). All of the antibodies were obtained from BioLegend (San Diego, CA, USA).

### 2.4. Hematoxylin and Eosin (H&E) Staining

According to a previous study [31], after fixation with 4% paraformaldehyde and dehydration, the tissues were embedded in paraffin and were exposed to gradient ethanol. The specimens were then cut into standard 5-μm sections, and were stained using hematoxylin and eosin (H&E), and examined using a microscope (Eclipse E100; Nikon, Tokyo, Japan).

### 2.5. Immunohistochemistry (IHC)

The protocol was adjusted based on our previous study [32]; 5-µm thick paraffin-embedded sections of the tumor were deparaffinized and rehydrated with paraffin, blocked with 3% H_2_O_2_ for 10 min, sealed with 3% BSA (G5001; Servicebio, Wuhan, China) for 30 min, and were then incubated overnight at 4 °C with primary antibodies against CD3e (#GB13014), CD4 (#GB13064-2), CD8 (#GB13429), CD20 (#GB11540), and IL-2 (#GB11114). The sections were incubated with goat anti-rabbit IgG(H+L) (peroxidase/HRP-conjugated; #G1213). After staining with diaminobenzidine (#G1212; Servicebio), the slices were re-stained with hematoxylin for 3 min, dehydrated, and blocked. The slides were examined using a microscope (Nikon DS-U3, Nikon). All of the antibodies were obtained from Servicebio.

### 2.6. Gut Microbiota Analysis

As in our previous study [33], the total microbial genomic DNA samples were isolated from cecal content (*n =* 4) using an OMEGA Soil DNA Kit (#M5635-02; Omega Bio-Tek, Norcross, GA, USA), and extraction was performed using a DNeasy PowerSoil Kit (QIAGEN, Inc., Venlo, The Netherlands). The quantity and quality of the extracted DNAs were measured, using a NanoDrop ND-1000 spectrophotometer (Thermo Fisher Scientific) and agarose gel electrophoresis, respectively. The V3–V4 regions of the bacterial 16S rRNA gene were amplified through polymerase chain reaction (PCR) with primers 338F (5′-ACTCCTACGGGAGGCAGCA-3′) and 806R (5′-GGACTACHVGGGTWTCTAAT-3′). The PCR products were purified, using Agencourt AMPure beads (Beckman Coulter, Indianapolis, IN, USA) and were quantified using a PicoGreen dsDNA Assay Kit (Invitrogen, Carlsbad, CA, USA). The amplicons were pooled at equal amounts, and the sequencing was performed on an Illumina MiSeq platform (Illumina, San Diego, CA, USA). The data were analyzed as previously described [34].

### 2.7. Metabolomics Analysis

Metabolomics analysis was performed on the serum samples (*n =* 4) which were added to a pre-cooled methanol/acetonitrile/water solution (2:2:1, *v*/*v*), followed by sonication at a low temperature for 30 min and centrifugation at −20 °C for 10 min; the supernatant was dried under vacuum. An aqueous acetonitrile solution (100 μL, acetonitrile: water = 1:1, *v*/*v*) was added, and after centrifugation, the supernatant was used for mass spectrometry analysis through ultra-high-performance liquid chromatography (1290 Infinity LC; Agilent Technologies, Santa Clara, CA, USA) coupled to a quadrupole time-of-flight (Q-TOF, TripleTOF 6600; Sciex, Framingham, MA, USA) mass spectrometer at Shanghai Applied Protein Technology (Shanghai, China). The detailed detection parameters were the same as those used in our previous study [35]. Univariate statistical analysis, multidimensional statistical analysis, differential metabolite screening, differential metabolite correlation analysis, and Kyoto Encyclopedia of Genes and Genomes (KEGG) pathway analyses were performed, as described previously [36].

### 2.8. Cytokine Detection

The spleen and tumor tissue samples were rinsed in ice-cold PBS and were homogenized in radioimmunoprecipitation assay buffer (#PC101; EpiZyme, Shanghai, China) containing a combination of protease and phosphatase inhibitors (#P002; NCM Biotech, Suzhou, China). The protein concentration was determined using an Omni-Easy™ Instant BCA Protein Assay Kit (#ZJ102; EpiZyme, Shanghai, China). The levels of IL-5 (#KE10018), IL-6 (#KE10007), IL-10 (#KE10008) (Proteintech, Chicago, IL, USA), arginine (Arg; #MM-0763M1; MEIMIAN, Shanghai, China), arginase I (Arg-1; #JL13668; JiangLai, Shanghai, China), nitric oxide (NO) assay kit (#S0021M; Beyotime, Shanghai, China), and IL-2 (#RK00007; Abclonal, Wuhan, China) were analyzed, using commercial ELISA kits according to the manufacturer’s instructions.

### 2.9. Western Blot

The tumor and spleen tissues were lysed, as described in Section 2.8. The proteins (40 μg) were electrophoresed using an Omni-Easy™ One-Step PAGE Gel Fast Preparation Kit (#PG212, EpiZyme), and were transferred to polyvinylidene difluoride membranes (0.45 μm; Merck, Darmstadt, Germany). After blocking in NcmBlot blocking buffer (#P30500; NCM Biotech) at 4 °C for 30 min, the membranes were incubated with primary antibodies (Table S1, Supplementary Materials) at 4 °C overnight. After washing with Tris-buffered saline containing 0.1% Tween-20, the membranes were incubated separately at 4 °C for 4 h with the appropriate horseradish peroxidase-conjugated secondary antibodies (Table S1, Supplementary Materials). The protein bands were visualized using electrochemiluminescence detection kits (Merck Millipore, Billerica, MA, USA) and a Tanon 5200 gel imaging system (Tanon Science & Technology, Shanghai, China). The band intensity was assessed using ImageJ software v1.8.0 (National Institutes of Health, Bethesda, MD, USA).

### 2.10. Statistical Analyses

One-way analysis of variance was performed, followed by post-hoc multiple comparisons (Tukey’s test) using DSS 25.0 software (version 25.0; IBM Corporation, Armonk, NY, USA). The statistical significance is reported at *p* < 0.05. The graphs were generated using GraphPad Prism 7.0 (GraphPad Software Inc., San Diego, CA, USA).

## 3. Results

### 3.1. Main IHS Compounds

The IHS contained 39% sugar, 15.3% protein, 2.65% polyphenols, 2.07% flavonoids, 1.34% saponin, 0.8% fat, 0.39% triterpenoids, 0.15% sterol, and 0.07% alkaloids (Table 1); it contained six minerals (Mn, Fe, Zn, Se, K, and Ca) and low concentrations of heavy metals. According to the standard GB2762-2017 in China, the content of the heavy metals (Pb, Hg, As, Cd, and Cr) was within the safety limits (Table 2).

### 3.2. IHS Restricts Tumor Growth in Apc^Min/+^ Mice

The IHS reduced the volume and abundance of colorectal tumors (Figure 1B) and colorectal indices (*p* < 0.05; Figure 1C) without influencing the organ indices (Figure S1A–E, Supplementary Materials) in the *Apc^Min/+^* mice. According to the H&E staining, the tumor cells in the mucosal layer of the colorectum of the *Apc^Min/+^* mice were heteromorphic, with a high nucleoplasm ratio and glandular tubular arrangement, all of which was reversed by IHS treatment (Figure 1D). Furthermore, the inflammatory cell infiltration in the heart and a large amount of hepatocyte hydropic degeneration with loose and lightly stained cytoplasm in the liver of *Apc^Min/+^* mice were ameliorated by IHS treatment (Figure S1F, Supplementary Materials).

### 3.3. IHS Alters the Gut Microbiota of Apc^Min/+^ Mice

**Figure 2:**
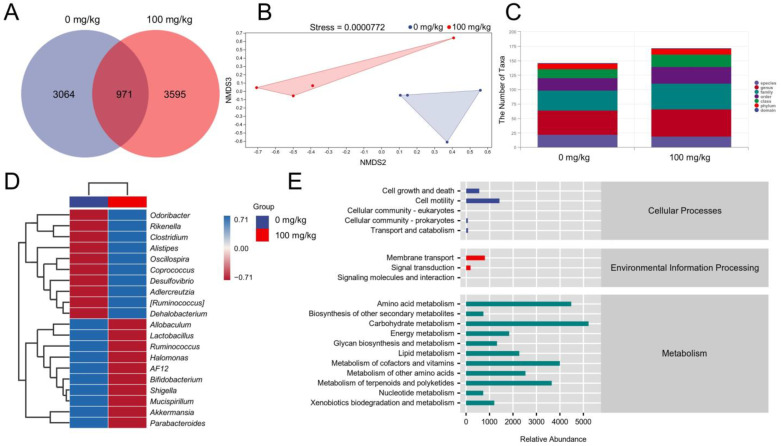
IHS modulates the gut microbiota of *Apc^Min/+^* mice. (**A**) Venn diagram; (**B**) Nonmetric multidimensional scaling of weighted UniFrac distances according to beta diversity analyses; (*n =* 4); (**C**) Analysis of the number of classification units; (**D**) Heatmap of gut microbes at the top 20 genus level based on relative abundance; (**E**) Predicted abundance graph of Kyoto Encyclopedia of Genes and Genomes (KEGG) secondary functional pathways; x-coordinate: relative abundance of functional pathways; y-coordinate: KEGG secondary functional pathways and the rightmost is the first level pathway to which the pathway belongs.

An imbalance in the gut microbiota is closely associated with the development of colorectal cancer [5]. According to the produced Venn diagram, 7630 OTUs were detected in the two experimental groups; however, only 971 (12.73%) OTUs occurred in both of the groups (Figure 2A), suggesting significant differences in the composition of the microbial communities. A nonmetric multidimensional scaling plot (beta diversity) obtained by the weighted UniFrac distance matrix (stress value = 0.0000772) showed a significantly different clustering of gut microbiota between the vehicle- and IHS-treated mice (Figure 2B). The IHS administration increased the abundance of *Oscillospira*, *Odoribacter*, *Rikenella*, *Dehalobacterium*, and *Coprococcus* and reduced the abundance of *Allobaculum* and *Mucispirillum* (Figure 2C,D; Table S2, Supplementary Materials). According to an analysis based on the KEGG database of metabolic pathways, the amino acid metabolism pathway was involved in IHS-mediated anti-CRC activity (Figure 2E).

### 3.4. IHS Influences Serum Metabolites in Apc^Min/+^ Mice

**Figure 3:**
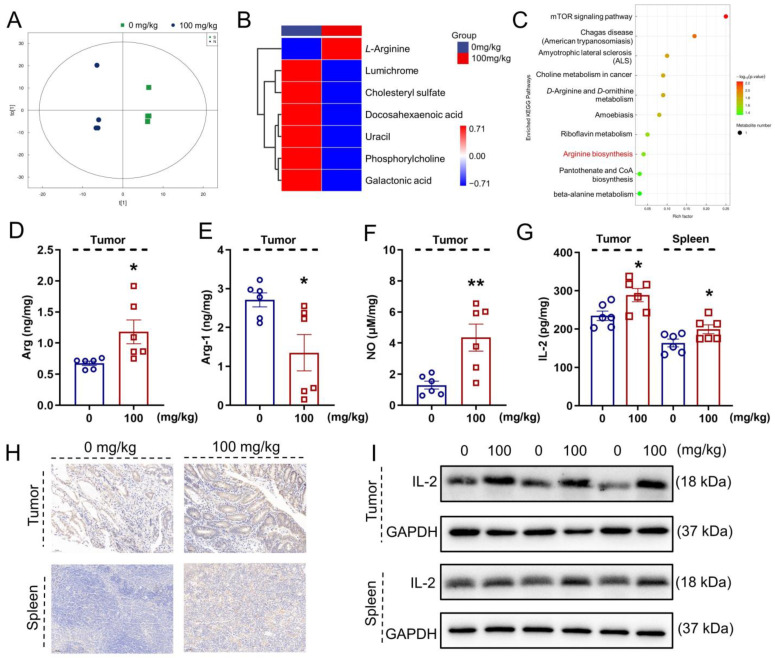
IHS regulates serum metabolites of *Apc^Min/+^* mice. (**A**) OPLS-DA was used to analyze differences in serum metabolites between vehicle- and IHS-treated mice; (**B**) Cluster heat map of differential metabolites; (**C**) KEGG enrichment pathway bubble map. IHS increased the level of (**D**) Arg and (**F**) NO, and decreased the level of (**E**) Arg-1 in tumors, as analyzed using ELISA (*n =* 6); (**G**–**I**) IHS enhanced the expressions of IL-2 in tumors and in the spleen of *Apc^Min/+^* mice, as analyzed using (**G**) ELISAs (*n =* 6), (**I**) Western blotting, and (**H**) IHC (20 ×, 50 μm). Data were analyzed using a one-way ANOVA and are shown expressed as means ± SEM. * *p* < 0.05, ** *p* < 0.01 vs. vehicle-treated mice.

The metabolites in the blood are associated with the gut microbiota under various physiological or pathological conditions [37]. The orthogonal partial least squares discriminant analysis (OPLS-DA) showed that the metabolite profiles were completely clustered into two groups, suggesting significantly different serum metabolic profiles in each group (Figure 3A). OPLS-DA VIP > 1 and *p* < 0.05 were used to identify differential metabolites. Compared with the vehicle-treated *Apc^Min/+^* mice, the IHS treatment enhanced the level of *L*-arginine (*L*-Arg) and reduced the levels of docosahexaenoic acid, cholesteryl sulfate, phosphorylcholine, galactonic acid, lumichrome, and uracil in the serum (Figure 3B; Table S3, Supplementary Materials). According to the KEGG analysis, 10 potential targeted metabolism pathways were screened, of which *L*-Arg participated in six metabolic pathways (Figure 3C; Table S4, Supplementary Materials). *L*-Arg was a key metabolite identified among the differential metabolites. *L*-Arg is the precursor for the biosynthesis of NO [38], and Arg-1 limits the production of NO by the catabolism of Arg [39]. A high expression of IL-2 induced a high production of *L*-Arg and NO [40]. The IHS increased the levels of Arg (*p* < 0.05; Figure 3D) and NO (*p* < 0.01; Figure 3F) and reduced the levels of Arg-1(*p* < 0.05; Figure 3E) in tumors. The IHS increased the levels of IL-2 (*p* < 0.05; Figure 3G) in tumors and in the spleen of *Apc^Min/+^* mice. The increment in the levels of IL-2 by IHS in tumors and in the spleen of *Apc^Min/+^* mice was further confirmed by IHC (*p* < 0.01; Figure 3H; Figure S2A, Supplementary Materials), and Western blotting (*p* < 0.01; Figure 3I; Figure S2B, Supplementary Materials).

### 3.5. IHS Increases the Abundance of CD8^+^ T Cells

**Figure 4:**
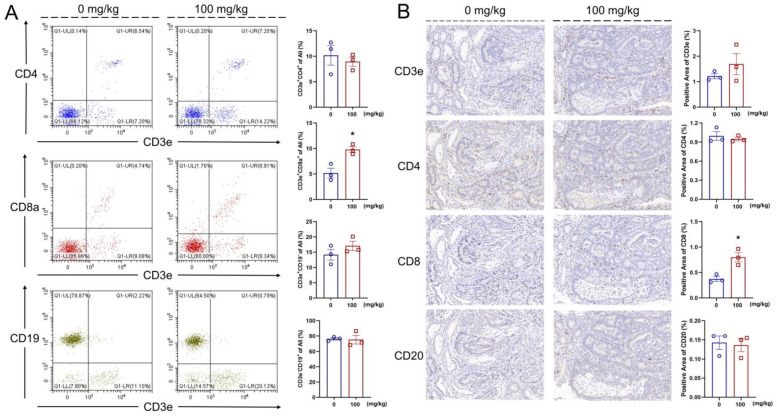
IHS enhanced the abundance of CD8^+^ T cell. IHS enhanced the abundance of CD3e^+^CD8a^+^ T cells and no significant change in the abundance of CD3e^+^CD4^+^, CD3e^+^CD19^−^, and CD3e^−^CD19^+^ cell in blood (**A**); IHS enhanced the positive area of CD8 and does not significantly change the positive area of CD3e, CD4, CD20 in tumor (**B**) (*n =* 3; 20×, 50 μm). Data were analyzed using a one-way ANOVA and are shown as means ± SEM. * *p* < 0.05, vs. vehicle-treated mice.

IL-2 stimulates the cytotoxic activity of CD8^+^ T cells by binding to the IL-2 receptor and activating the JAK/STAT signaling pathway [41]. In the IHS-treated mice, an increased abundance of the CD3e^+^CD8a^+^ cells (*p* < 0.05) was observed in the blood; however, no significant changes were found regarding the abundances of CD3e^+^CD19^−^, CD3e^−^CD19^+^, and CD3e^+^CD4^+^ cells in the blood (Figure 4A). The IHC showed that the IHS treatment increased the positive area of CD8 (*p* < 0.05) of tumor tissues, indicating an increase in the abundance of CD8^+^ cells in the tumor (Figure 4B); thus, IHS may mediate the tumor cell-killing effect by CD8^+^ T cells through increasing their abundance.

### 3.6. IHS Regulates JAK/STAT Signaling in Apc^Min/+^ Mice

**Figure 5:**
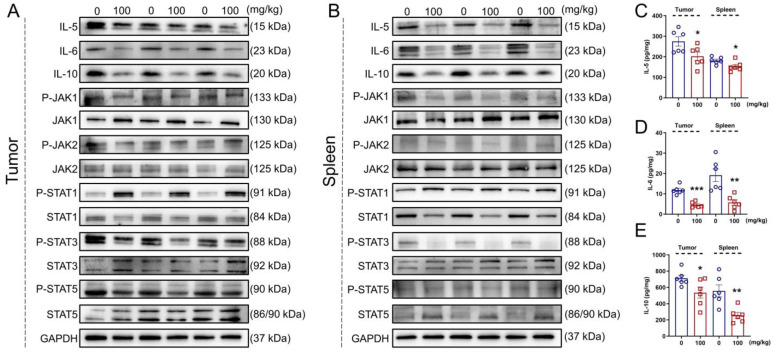
IHS inhibits JAK/STAT3/IL6 signaling in *Apc^Min/+^* mice. IHS decreased the protein levels of IL-5, IL-6, IL-10, P-JAK1/JAK1, P-JAK2/JAK2, P-STAT3/STAT3, and P-STAT5/STAT5 and increased the level of P-STAT1/STAT1, as detected through Western blotting of tumor (**A**) and spleen tissue (**B**) of *Apc^Min/+^* mice. IHS decreased the levels of (**C**) IL-5, (**D**) IL-6, and (**E**) IL-10 in tumor and spleen tissue of *Apc^Min/+^* mice, as detecting through ELISA. Data were analyzed using a one-way ANOVA and are shown as means ± SEM. * *p* < 0.05, ** *p* < 0.01 and *** *p* < 0.001 vs. vehicle-treated group.

In both the tumor and spleen of the *Apc^Min/+^* mice, IHS strongly suppressed the expression of IL-5 (*p* < 0.05; Figure S3A, Supplementary Materials), IL-6 (*p* < 0.05; Figure S3B, Supplementary Materials), IL-10 (*p* < 0.05; Figure S3C, Supplementary Materials), P-JAK1 (*p* < 0.05; Figure S3D, Supplementary Materials), P-JAK2 (*p* < 0.05; Figure S3E, Supplementary Materials), P-STAT3 (*p* < 0.05; Figure S3G, Supplementary Materials), and P-STAT5 (*p* < 0.05; Figure S3H, Supplementary Materials) and enhanced the P-STAT1 expression (*p* < 0.01; Figure 5A,B; Figure S3F, Supplementary Materials). The reduction in IL-5 (*p* < 0.05; Figure 5C), IL-6 (*p* < 0.01; Figure 5D), and IL-10 (*p* < 0.05; Figure 5E) in the tumor and spleen of the IHS-treated *Apc^Min/+^* mice was further confirmed through ELISAs.

## 4. Discussion

Here, we report for the first time the inhibitory effects of IHS on tumor growth in *Apc^Min/+^* mice, which was related to its regulation of gut microbiota-mediated immune function, especially through the regulation of the abundance of CD8^+^ T cells. Encouragingly, the tumor cells in the mucosal layer of the colorectum of *Apc^Min/+^* mice were heteromorphic, with a high nucleoplasm ratio and glandular tubular arrangement, all of which were reversed by IHS.

The nutritional composition of IHS was systematically analyzed, and the content of dietary fiber, polyphenols, and triterpenoids was assessed, which provided a basis for identifying its anti-CRC activity. A low intake of dietary fiber is one of the main factors of CRC susceptibility [4]. Natural polyphenols are a rich source of natural antioxidants, prebiotics, and dietary polyphenols, which serve as immunomodulators and can inhibit the etiology and pathogenesis of CRC [42]. Additionally, triterpenoids can inhibit the proliferation of tumor cells by inhibiting glycolysis, thus acting as anti-CRC agents [43].

The dysbiosis of the human gut microbiota is closely associated with the development of CRC [5], as evidenced by the alteration of *Ruminococcus*, *Clostridium*, *Coprococcus*, *Oscillospira*, *Odoribacter*, *Bifidobacterium*, and *Lactobacillus* in patients with CRC [44,45,46]. Corresponding changes were observed in *Apc^Min/+^* mice and were improved after IHS administration. *Ruminococcus* can degrade natural polysaccharides that are not digestible by humans [47,48]. *Bifidobacterium* and *Lactobacillus* help produce lactate from carbohydrate substrates [48]. However, an accumulation of *D*-lactate is life-threatening in cases with short bowel syndrome [49]. The acidification caused by lactate increases the expression of Arg-1, which hydrolyzes *L*-Arg in the macrophages [50,51]. A depletion of *L*-Arg leads to impaired CD3 ζ-chain expression, especially in CD8^+^ T cells, resulting in cell growth inhibition [52]. The IHS increased the levels of *L*-Arg in the tumor and spleen of the *Apc^Min/+^* mice, suggesting an important role of the gut microbiota regarding the anti-CRC effects of IHS.

Furthermore, low levels of Arg have been observed in patients with CRC [53], and *L*-Arg metabolism can regulate the immune responses [54]. *Clostridium* converts Arg to ornithine, which facilitates Arg transport [55]. *L*-Arg is a precursor of NO synthesis [56], which is associated with a strong induction of IL-2 in humans and other species [57]. In a feedback loop, the high expression of IL-2 induces increased *L*-Arg and NO production [40], and *Lactobacillus* significantly induces IL-2 [58]. IL-2 promotes CD8^+^ T cells differentiation and expansion during immune regulation [59], which contributes to the effective restoration of T cell functioning during CRC [60]. The cytokines are key components of the immune barrier, some of which act on epithelial tissues, especially in the gut [61]. The CD8^+^ T cells inhibit IL-5 synthesis to reduce the eosinophil infiltration in models of experimental lung disease [62]. IL-6 induces strong immunosuppression in the CRC microenvironment by recruiting immunosuppressive cells, impairing T cell infiltration, and reducing the abundance of CD8^+^ T cells [63]. Additionally, IL-10 suppresses the immune response by inhibiting the T cell-proliferation and inducing T cell depletion [64]. A high abundance of CD8^+^ T cells was confirmed in the blood and at the tumor sites of the IHS-treated *Apc^Min/+^* mice. Taken together, the CD8^+^ T cells are involved in IHS-mediated anti-CRC processes related to the regulation of immune responses [7].

The JAK/STAT3 pathway is closely associated with the CD8^+^ T cell differentiation and maturation [65,66], and is a potential target for the CRC treatment [19]. IL-2 can activate several downstream signaling molecules, including the JAKs, by binding to their receptors [20]. The JAKs mediate the recruitment of STAT1, 3, and 5 [20]. The sustained activation of STAT3 increases the tumor cell proliferation, survival, and invasion, thereby accelerating the tumor formation process [67]. IL-6, a pleiotropic cytokine, can promote Arg-1 expression through STAT3 [68,69]. In contrast, IL-6 activates the JAK/STAT signaling through glycoprotein 130 [22]. The IHS inhibits the activation of JAK/STAT and affects the abundance of the CD8^+^ T cells, regulates the immune response, and inhibits CRC development.

This study has some limitations. Owing to production restrictions, IHS is currently difficult to commercialize, and little research has been conducted on its pharmaceutical effects. IHS comprises a complex mixture of nutrients, thus further research is required to identify its active substances. Moreover, different from our study, some of the research performed the metataxonomics in dirty colon tissue and the metabolites in the clean colon tissue of CRC mice [70,71], which should be followed in our future studies.

## 5. Conclusions

In this study, we confirmed for the first time the inhibitory effects of IHS on CRC development in *Apc^Min/+^* mice, and through combining gut microbiota analyses and serum metabolomics, this effect was confirmed to be associated with the regulation of CD8^+^ T cell abundance through JAK/STAT signaling.

## Figures and Tables

**Table 1:** The general nutritional composition of IHS.

Compounds	Contents (%)
Protein	15.3
Fat	0.8
Total sugar	39
Total dietary fiber	70.23
Total sterol	0.15
Total alkaloid	0.07
Total triterpenoids	0.39
Total flavonoids	2.07
Total saponin	1.34
Total polyphenols	2.65

**Table 2:** The heavy metals and metal composition of IHS.

	Compounds	Contents (mg/kg)
Heavy metals	Lead (Pb)	0.368
	Mercury (Hg)	0.0189
	Arsenic (As)	0.117
	Cadmium (Cd)	0.064
	Chromium (Cr)	0.0939
	Copper (Cu)	9.46
Metals	Manganese (Mn)	2.4
	Iron (Fe)	27.9
	Zinc (Zn)	11.4
	Selenium (Se)	0.0823
	Potassium (K)	7.18 × 10^3^
	Calcium (Ca)	124
	Sodium (Na)	UD *

* UD: undetected; the detection limit was 3 mg/kg.

## Data Availability

The data sets and materials supporting the conclusions of this study are included within the article and its Supplementary Materials.

## Supplementary Materials

The following supporting information can be downloaded at: https://www.mdpi.com/article/10.3390/nu14163299/s1, Figure S1: Observation of mice organ index and viscera (heart, liver, spleen, lungs, and kidney) H&E staining; Figure S2: IHS regulates IL-2 levels in tumors and spleens of *Apc^Min/+^* mice; Figure S3: Quantification of protein signals in Figure 5A,B normalized to GAPDH; Table S1: Details of antibodies used in Western blot; Table S2: Relative abundance of top 20 genera; Table S3: Significantly different metabolites; Table S4: Annotation of the KEGG pathway for significantly different metabolites.
