# Supplementary material for: Anti-Colorectal Cancer Effects of Inonotus hispidus (Bull.: Fr.) P. Karst. Spore Powder through Regulation of Gut Microbiota-Mediated JAK/STAT Signaling
Source: Nutrients. 2022 Aug 12;14(16):3299. doi: 10.3390/nu14163299 (PMC9415721; doi:10.3390/nu14163299)
Supplement: Supplementary file 1 [file nutrients-14-03299-s001.zip › nutrients-1823146-supplementary.pdf]

Supplementary Date

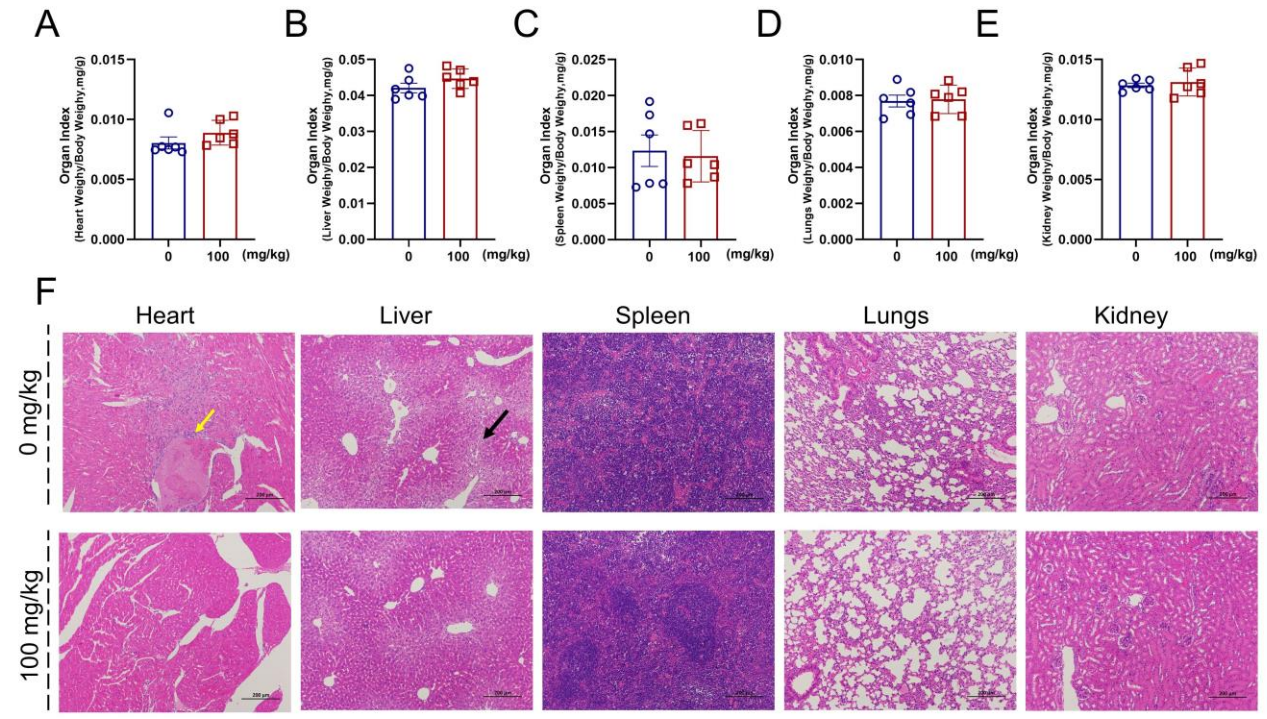

Figure S1. Observation of mice organ index and viscera (heart, liver, spleen, lungs, and kidney) H&E staining. *Inonotus hispidus* (Bull.:Fr.) P. Karst. spore powder (IHS) shows no significant effects on organ indexes of (A) heart, (B) liver, (C) spleen, (D) lung, and (E) kidney. (F)Vehicle-treated mice with inflammatory cell infiltration in the heart (yellow arrow) and a large amount of hepatocyte hydropic degeneration with loose and lightly stained cytoplasm in the liver (black arrow), and no significant changes on other organ structures including spleen, lung, and kidney analyzed by H&E staining (200×, 100 μm). The data are shown as means ± SEM.

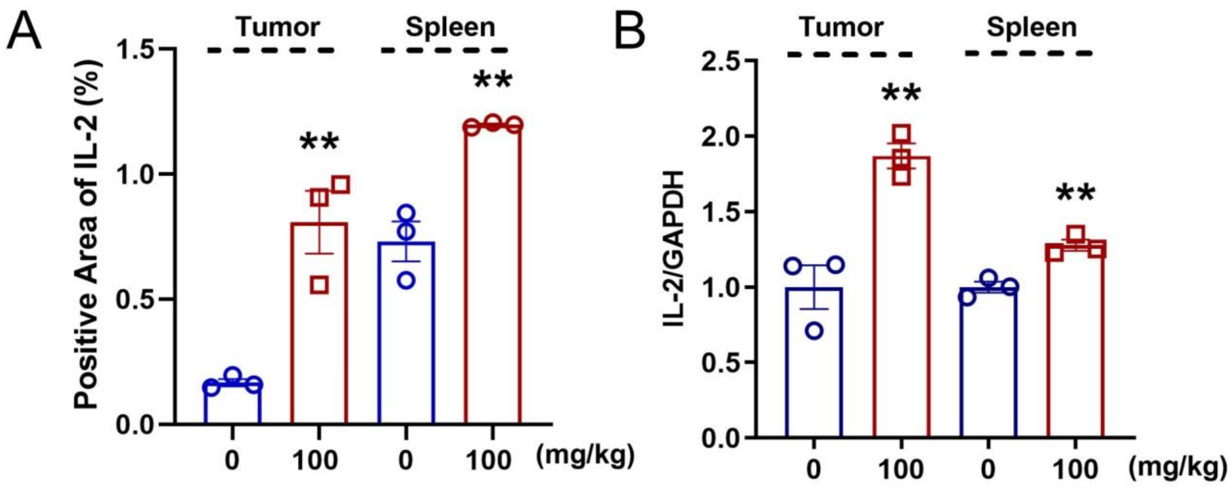

Figure S2. IHS regulates IL-2 levels in tumors and spleens of *Apc*$^{Min/+}$ mice. (A) Quantification of immunohistochemistry in Figure 3H. (B) Quantification of protein signals in Figure 3I normalized to GAPDH. The data are shown as means ± SEM. ***p* < 0.01 *vs*. vehicle-treated group.

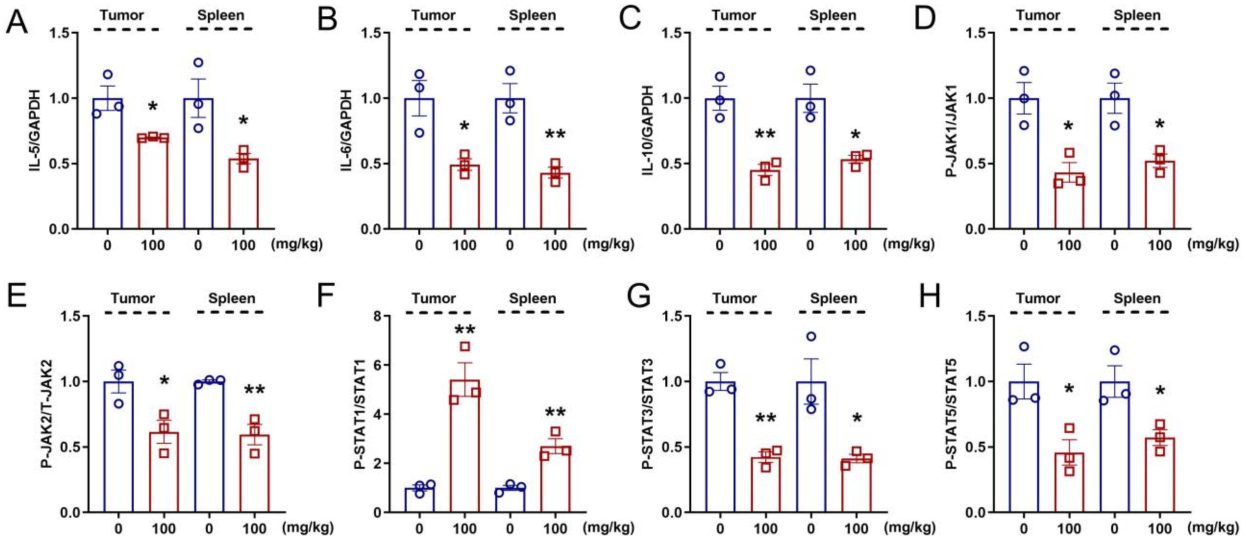

Figure S3. Quantification of protein signals in Figure 5A, B normalized to GAPDH. The levels of (A) IL-5, (B) IL-6, (C) IL-10, (D) P-JAK1/JAK1, (E) P-STAT1/STAT1, (F)P-JAK2/JAK2, (G)P-STAT3/STAT3, and (H) P-STAT5/STAT5 in tumor and spleen tissue of *Apc*$^{Min/+}$ mice. The data are shown as means ± SEM. **p* < 0.05, ***p* < 0.01 *vs*. vehicle-treated group.

Table S1. Details of antibodies used in Western blot.

Antibody	Molecular weight	Catalog number	Dilution	Company	Area
IL-2	18kDa	A16317	1:1000	ABclonal	Wuhan, China
IL-5	15kDa	A7690	1:1000	ABclonal	Wuhan, China
IL-6	23kDa	A11115	1:1000	ABclonal	Wuhan, China
IL-10	20kDa	A2171	1:1000	ABclonal	Wuhan, China
Phospho-JAK1 (Tyr1022, Tyr1023)	133kDa	44-422G	1:1000	Invitrogen	Waltham, MA, USA
JAK1 [EPR349(N)]	130kDa	ab133666	1:1000	Abcam	Cambridge, MA, USA
JAK2 (phospho Y1007 + Y1008)	125kDa	ab68268	1:5000	Abcam	Cambridge, MA, USA
JAK2	125kDa	3230s	1:1000	Cell Signaling Technology	Danvers, MA, USA
Phospho-STAT1-Y701	91kDa	AP0054	1:1000	ABclonal	Wuhan, China
STAT1	84kDa	A0027	1:1000	ABclonal	Wuhan, China
STAT3 (phospho Y705)	88kDa	ab76315	1:1000	Abcam	Cambridge, MA, USA
STAT3	92kDa	06-596	1:1000	MILLIPORE	Darmstadt, Germany
Phospho-STAT5A-Y694	90kDa	AP0758	1:1000	ABclonal	Wuhan, China
STAT5A	86/90kDa	A11779	1:1000	ABclonal	Wuhan, China
GAPDH	37kDa	E-AB-20032	1:2000	Elabscience	Wuhan, China
Goat Anti-Rabbit (H+L)	/	E-AB-1003	1:4000	Elabscience	Wuhan, China
Goat-Anti-Mouse (H+L)	/	E-AB-1001	1:4000	Elabscience	Wuhan, China

Table S2. Relative abundance of top 20 genera.

Sample	Vehicle-treaded mice	IHS-treaded mice
Oscillospira	0.097955	0.169959
Allobaculum	0.082144	0.059823
Lactobacillus	0.114726	0.024105
Shigella	0.110002	0.004176
Akkermansia	0.031809	0.027953
Parabacteroides	0.020029	0.017694
Rikenella	0.009729	0.014764
Mucispirillum	0.012772	0.011265
Odoribacter	0.008289	0.010785
Ruminococcus	0.011723	0.00439
[Ruminococcus]	0.006066	0.009914
Desulfovibrio	0.004181	0.010036
Clostridium	0.00383	0.006955
Adlercreutzia	0.001485	0.004333
Dehalobacterium	0.002346	0.003399
Coprococcus	0.001195	0.003756
Halomonas	0.001968	0.000947
Bifidobacterium	0.00106	0.000879
AF12	0.001076	0.000843
Alistipes	0.000426	0.000742

Data are presented as the mean.

Table S3. Significantly different metabolites.

**Adduct**	**Name**	**VIP**	***p*-value**	**Mean of Vehicle-treaded mice**	**Mean of IHS-treaded mice**
(M+H)+	*L*-Arginine	3.019092	0.004253	243848.3	459369.6
(M+H)+	Phosphorylcholine	4.27844	0.019356	973718.3	379996.5
(M+H)+	Lumichrome	1.161879	0.048048	120907.2	70138.07
(M-H)-	Galactonic acid	1.169921	0.021808	106910	60121.02
(M-H)-	Uracil	5.339849	0.032693	2482826	1438143
(M-H)-	Cholesteryl sulfate	1.191214	0.035847	97470.28	46365.46
(M-H)-	Docosahexaenoic acid	10.15721	0.043834	9970878	5480321

Adduct: Adduct ion information for compounds;

VIP: Variable importance for the projection of orthogonal partial least squares discriminant analysis (OPLS-DA).

Table S4. Annotation of the KEGG pathway for significantly different metabolites.

Involved mechanism	Metabolite
mTOR signaling pathway	*L*-Arginine
Chagas disease (American trypanosomiasis)	*L*-Arginine
Amyotrophic lateral sclerosis (ALS)	*L*-Arginine
D-Arginine and D-ornithine metabolism	*L*-Arginine
Choline metabolism in cancer	Phosphorylcholine
Amoebiasis	*L*-Arginine
Riboflavin metabolism	Lumichrome
Arginine biosynthesis	*L*-Arginine
Pantothenate and CoA biosynthesis	Uracil
beta-Alanine metabolism	Uracil
